# Interfering Satellite RNAs of *Bamboo mosaic virus*

**DOI:** 10.3389/fmicb.2017.00787

**Published:** 2017-05-04

**Authors:** Kuan-Yu Lin, Na-Sheng Lin

**Affiliations:** Institute of Plant and Microbial Biology, Academia SinicaTaipei, Taiwan

**Keywords:** interfereing, satellite RNA, BaMV, competition, RNA silencing

## Abstract

Satellite RNAs (satRNAs) are sub-viral agents that may interact with their cognate helper virus (HV) and host plant synergistically and/or antagonistically. SatRNAs totally depend on the HV for replication, so satRNAs and HV usually evolve similar secondary or tertiary RNA structures that are recognized by a replication complex, although satRNAs and HV do not share an appreciable sequence homology. The satRNAs of *Bamboo mosaic virus* (satBaMV), the only satRNAs of the genus *Potexvirus*, have become one of the models of how satRNAs can modulate HV replication and virus-induced symptoms. In this review, we summarize the molecular mechanisms underlying the interaction of interfering satBaMV and BaMV. Like other satRNAs, satBaMV mimics the secondary structures of 5′- and 3′-untranslated regions (UTRs) of BaMV as a molecular pretender. However, a conserved apical hairpin stem loop (AHSL) in the 5′-UTR of satBaMV was found as the key determinant for downregulating BaMV replication. In particular, two unique nucleotides (C^60^ and C^83^) in the AHSL of satBaMVs determine the satBaMV interference ability by competing for the replication machinery. Thus, transgenic plants expressing interfering satBaMV could confer resistance to BaMV, and interfering satBaMV could be used as biological-control agent. Unlike two major anti-viral mechanisms, RNA silencing and salicylic acid-mediated immunity, our findings in plants by *in vivo* competition assay and RNA deep sequencing suggested replication competition is involved in this transgenic satBaMV-mediated BaMV interference. We propose how a single nucleotide of satBaMV can make a great change in BaMV pathogenicity and the underlying mechanism.

## Introduction

Satellite RNAs (satRNAs) are short RNA molecules that share no or little sequence homology to their cognate helper virus (HV) but totally depend on the HV for replication, encapsidation and efficient movement (Hu et al., 2009; Briddon et al., 2012). The homology sequence between satRNAs and their HVs often resides at the 5′ and 3′ regions. Usually conserved secondary structure functions such as the *cis*-acting element are essential for replicase recognition acting as mimicry of molecular pretenders at the 5′ and 3′ regions. SatRNA mimicry is mostly conserved in higher-order RNA structures. As well, satRNAs may adopt different mimicry at different stages of virus infection such as replication and translation (Huang et al., 2010).

Satellite RNAs have attracted great interest in the past decades because they can modulate symptoms caused by their HVs (Palukaitis, 1988; Li and Simon, 1990; Collmer and Howell, 1992; Hsu et al., 1998), alter HV RNA accumulation (Buzayan et al., 1986; Gal-On et al., 1995; Hsu et al., 1998), enhance HV movement (Zhang and Simon, 2003; Simon et al., 2004) and in at least one case, affect the infection cycle of their HV, for example, during insect transmission (Robinson et al., 1999; Taliansky et al., 2000). One of the most fascinating characteristics of satRNAs is their interference ability. There are many cases of symptom-attenuating satRNAs, such as satRNAs of isolates of the species *Bamboo mosaic virus* (BaMV), *Cucumber mosaic virus* (CMV), *Peanut stunt virus* (PSV), *Grapevine fanleaf virus, Artichoke mottled crinkle virus, Cymbidium ringspot virus* (CymRSV), *Tobacco ringspot virus* (TobRSV), and *Groundnut rosette virus* (GRV) (Roossinck et al., 1992; Simon et al., 2004).

Satellite RNAs of BaMV (satBaMVs) are well studied. Natural isolates of satBaMVs have been collected from BaMV-infected symptomatic bamboo plants worldwide to analyze the genetic evolution and phylogeny of satBaMVs (Liu et al., 1997; Wang et al., 2014). The mimicry of satBaMVs among the 5′- and 3′-untranslated regions (UTRs) have been investigated thoroughly (Annamalai et al., 2003; Huang et al., 2009), and the biological function of satBaMV-encoded protein elucidated its role in satBaMV replication (Lin et al., 1996), movement (Vijayapalani et al., 2006, 2012; Chang et al., 2016) and interference in BaMV replication (Hsu et al., 2006).

In this review, we focus on studies of interfering satBaMVs and a possible mechanism of satBaMVs interfering in BaMV infection.

## Bamv and its Associated SatBaMVs

**FIGURE 1:**
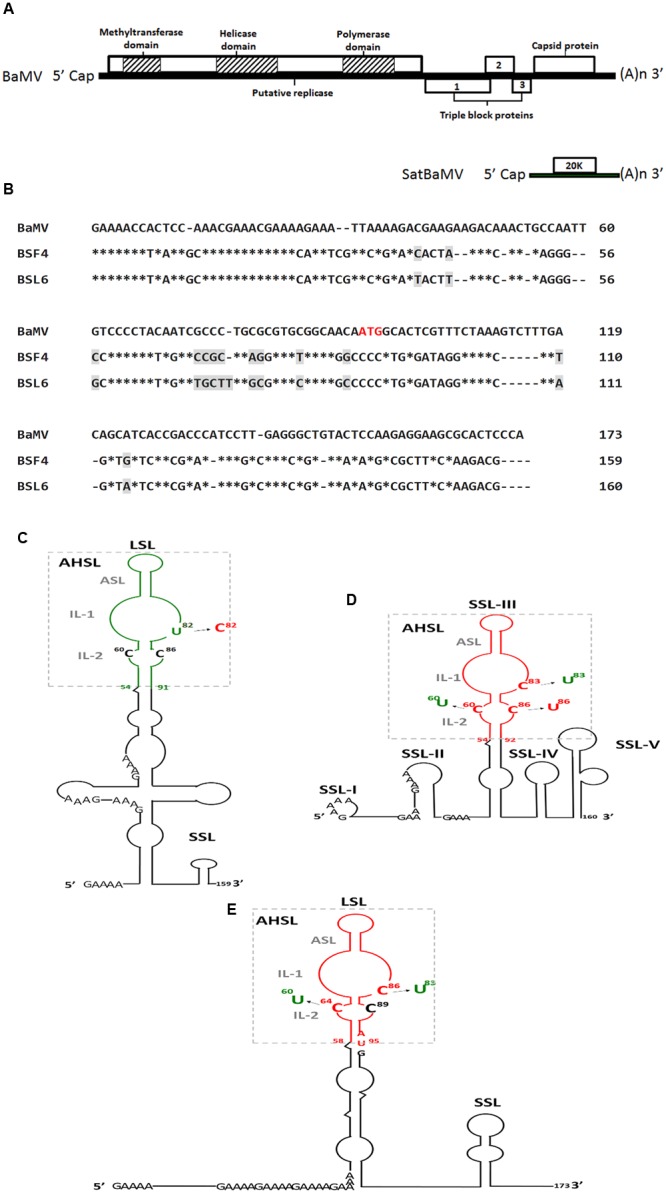
**Genome map of satellite *Bamboo mosaic virus* (satBaMV) and BaMV (A)**, sequence alignment of BaMV, BSF4, and BSL6 5′-UTR **(B)** and secondary structures of 5′-UTR of satBaMV, BSF4 **(C)** and BSL6 **(D)** and 5′-termini of BaMV **(E)** and their derived mutants. ^∗^indicates identical nucleotide. Different nucleotides between BSF4 and BSL6 sequence are marked by gray shade. The apical hairpin stem loop (AHSL) structures of satBaMV and BaMV are boxed, and all contain an apical stem loop (ASL) and two internal loops (IL-1 and IL-2). The common GAAA(A) repeats in the 5′-UTRs are indicated. The AUG sequence indicates the start codon of the BaMV open reading frame 1 (ORF1). Green and red indicate the non-interfering and interfering type. LSL, large stem loop; SSL, small stem loop.

*Bamboo mosaic virus* is a single-stranded positive-sense RNA virus containing five open reading frames (ORFs) that belongs to the genus *Potexvirus* of the family *Alphaflexiviridae* (Lin et al., 1994). ORF1 encodes a replicase-related protein with three functional domains for BaMV replication: methyltransferase (Li et al., 2001a; Huang et al., 2004), helicase (Li et al., 2001b) and RNA-dependent RNA polymerase (RdRp) (Li et al., 1998). ORF2 to four encode triple gene block proteins, which are three overlapping proteins essential for BaMV movement (Wung et al., 1999; Lin et al., 2004, 2006). ORF5 encodes a coat protein (CP) for BaMV encapsidation, movement (Lee et al., 2011) and symptom formation (Lan et al., 2010) (**Figure 1A**).

*Bamboo mosaic virus* causes mosaic symptoms on infected bamboo leaves and infects at least 13 economically important bamboo species in Taiwan (Lin et al., 1993). In BaMV-infected bamboo, small single-stranded positive-sense RNA molecules that share no sequence homology with BaMV but replicate and encapsidate associated BaMV are defined as satBaMVs (Lin and Hsu, 1994). SatBaMV is the only potexvirus-associated satRNA. It is a 836-nt linear RNA molecule that encodes a 20-kDa non-structural protein (P20) flanked by a 159-nt 5′-UTR and 125-nt 3′-UTR (Lin and Hsu, 1994; **Figure 1A**). P20 is not essential for satBaMV replication (Lin et al., 1996), but it preferentially binds to satBaMV RNA (Tsai et al., 1999). However, P20 is necessary for satBaMV long-distance transport in BaMV–co-infected *Nicotiana benthamiana* (Vijayapalani et al., 2006, 2012; Chang et al., 2016). In the absence of BaMV, satBaMV RNA could undergo autonomous long-distance movement *in planta* (Chang et al., 2016).

Three phylogenetic satBaMV groups were classified from natural satBaMV isolates derived from 10 infected bamboo species in different locations of Taiwan, Hainan Island of China and Delhi, India (Liu et al., 1997; Yeh et al., 2004; Wang et al., 2014). Clade I contains all other satBaMVs except most of those isolated from Ma bamboo (*Dendrocalamus latiflorus* Munro) and all populations from *Bambusa vulgaris*. All satBaMVs in clades II and III are derived almost entirely from Ma bamboo from the Taipei Botanical Garden in Taiwan and *B. vulgaris* in India, respectively (Wang et al., 2014).

Sequence analysis of satBaMV isolates showed a hypervariable region with the greatest sequence variation in the satBaMV 5′-UTR but a conserved secondary RNA structure (Yeh et al., 2004). SatBaMV is totally dependent on BaMV for replication and encapsidation (Lin and Hsu, 1994). Therefore, 5′- and 3′-UTRs of satBaMV evolved similar RNA secondary structures and functional RNA elements with BaMV to recruit the RdRp encoded by BaMV for replication. These features include GAAA(A) repeats at the 5′-UTR and conserved hexanucleotides (ACCUAA) and polyadenylation signals (AAUAAA) at the 3′-UTR (Lin and Hsu, 1994; Lin et al., 1994). As well, the secondary structures of the satBaMV 3′-UTR contain two small stem-loops (SLA and SLB) and one large stem-loop (SLC) that are similar to the domains B, C, and D of the BaMV 3′-UTR, respectively (Cheng and Tsai, 1999; Huang et al., 2009). One of the alluring properties of satBaMVs is that some natural satBaMV isolates feature antagonistic ability against BaMV replication (Hsu et al., 1998, 2006). However, interfering satBaMVs isolated from different bamboo species and locations are not grouped in the same phylogenetic clades (Yeh et al., 2004). The mechanisms underlying satRNA-mediated HV interference is fascinating, but most cases have not been clearly demonstrated.

## The Determinant of SatBaMV Interference Resides in the 5′-Utr Apical Hairpin Stem Loop (AHSL)

Two satBaMV isolates, BSF4 and BSL6, exhibit different phenotypes in *N. benthamiana* co-infected with BaMV and satBaMV (Hsu et al., 1998). Attenuated BaMV-induced symptoms were found associated with reduced BaMV level (Hsu et al., 1998). The sequence of the BSF4 and BSL6 5′-UTR shares 92% identity, with only 13 mismatches (**Figure 1B**), but the secondary structures greatly differed, as revealed by enzymatic probing with RNases A, T1, T2, and V1. The secondary structures of the non-interfering BSF4 5′-UTR contain a large stem loop (LSL) and a small stem loop (SSL) (**Figure 1C**; Annamalai et al., 2003), whereas the interfering BSL6 5′-UTR contains five SSLs (**Figure 1D**; Chen et al., 2007). However, the 5′-UTR hypervariable region of both BSF4 (in LSL) and BSL6 (SSL-III) features a conserved apical hairpin stem loop (AHSL) structure including two internal loops (ILs; IL-1 and I-2) (**Figures 1C,D**; Annamalai et al., 2003; Chen et al., 2007). *In silico* secondary structure prediction of the 5′-UTR of natural satBaMV isolates by MFOLD revealed that most of the analyzed satBaMV isolates retained an identical AHSL structure despite their grouping into different phylogenetic clades (Yeh et al., 2004). Moreover, the RNA sequence in the AHSL region of BSF4 and BSL6 is interchangeable, and chimeric satBaMVs can replicate to a similar level as BSF4 and BSL6 when co-infected with BaMV in *N. benthamiana* protoplasts, so maintaining a conserved AHSL structure but not the sequence itself is essential for satBaMV replication (Yeh et al., 2004).

To elucidate the determinant of BSL6 interference of both BaMV-induced symptoms and BaMV level, chimeric satBaMV mutants with different combinations of BSF4 and BSL6 between the 5′-UTR, most coding regions of P20 and the 3′-UTR were investigated. All mutants containing the 5′-UTR of BSL6 could reduce BaMV level in both positive (+) and negative (-) strands without altering satBaMV level in *N. benthamiana* protoplasts and caused symptomless infection in *N. benthamiana* plants (Hsu et al., 2006). Moreover, both a BSL6 mutant expressing the truncated form of P20 and a frameshift mutant could reduce BaMV level, so P20 is not required for BSL6-mediated BaMV interference (Hsu et al., 2006). Furthermore, the BSL6 5′ UTR alone was sufficient to interfere with (+)- and (-)-strand BaMV level and BaMV-caused symptoms when expressed in a BaMV vector driven by a sub-genomic promoter in *Chenopodium quinoa* (Hsu et al., 2006). Thus, the BSL6 5′-UTR is the determinant of the interference in BaMV replication and the interference is independent of P20 translation.

On further analyzing the RNA secondary structure of natural satBaMV isolates, an identical AHSL structure was found shared by all natural interfering satBaMVs. SatBaMV mutants that swap the AHSL region of BSF4 and BSL6 revealed that the AHSL in the 5′-UTR is the determinant of satBaMV-mediated BaMV interference (Hsu et al., 2006). To further clarify whether the structure or sequence of AHSL is more important for BSL6-mediated interference, BSL6-derived mutants with disrupted AHSL structure or only sequence substitution were used to test BaMV interference. On co-inoculation with BaMV, all mutants with disrupted AHSL structure lost the ability to reduce BaMV level. Moreover, an identical AHSL structure with the sequence (^81^UGC^83^) in IL-1 was found in all natural interfering satBaMVs, whereas a less-conserved AHSL structure or identical AHSL structure but with different sequence (^81^UGU^83^) in IL-1 was found in non-interfering satBaMVs (Chen et al., 2007). Further analysis revealed that only one nucleotide substitution in U^82^ to C^82^ or C^83^ to U^83^ of BSF4 or BSL6, respectively, could change the phenotype (Chen et al., 2007). Another nucleotide C^60^ in IL-2 was also essential for BSL6-mediated interference. BSL6 C^60^U no longer reduced BaMV level (Chen et al., 2007). Thus, both the AHSL structure and two nucleotides C^60^ in IL-2 and C^83^ in IL-1 are essential for BSL6-mediated BaMV interference (**Figures 1C,D**).

Different hosts also feature a one-nucleotide substitution altering satRNA-induced symptoms or their ability to modulate HV-induced symptoms. With CMV in tomato, C^215^, C^286^ and A^330^ of WLM2-satCMV could independently affect necrosis induction with different CMV strains (Wu and Kaper, 1992; Sleat et al., 1994), and the satCMV Y-strain nucleotide 185/186 caused yellow mosaic symptoms in tobacco (Jaegle et al., 1990). For the PSV system, U^226^ and C^262^ determine symptom attenuation of PSV G-satRNA in tobacco (Naidu et al., 1992). These examples all imply that the pathogenicity of satRNAs result from the complex interaction between the host, HV and satRNAs.

However, only an approximate idea was proposed for the altered RNA secondary or tertiary structure being essential for necrosis induction of WLM2-satCMV caused by a single nucleotide change (Sleat et al., 1994). How a single nucleotide of satBaMV results in such a great change in the interference of BaMV-induced symptoms and BaMV replication is a fascinating mystery that remains to be solved.

## Conserved Secondary Structures in the 5′-Utr of BaMV and SatBaMV Are Involved in Competition for Replication Complexes

Because of the HV RdRp-dependent replication of satRNAs, competition for viral RdRp between satRNAs and HV was the first hypothesized and demonstrated as a mechanism for CMV and satCMV (Wu and Kaper, 1995). However, the authors used *in vitro* replication assay, which may not reflect the complex interaction between CMV and satCMV in co-infected plants (Wu and Kaper, 1995). Although many satRNAs reduce HV accumulation, no further studies have implied RdRp competition as the determinants of satRNAs-mediated interference. In contrast, a more complete analysis of the conserved secondary structure of BaMV and satBaMV implied that replication complex competition could be the major mechanism of satBaMV-reduced BaMV level.

First, interfering satBaMV is dominant among progeny populations in protoplasts with mixed-infected BaMV and non-interfering satBaMV (Chen et al., 2012). In addition, an *in vivo* replication system revealed that the replication efficiency is higher for BSL6 than BSF4 when the two are individually supported by abundant BaMV ORF1-encoded RdRp for replication in *N. benthamiana* protoplasts (Chen et al., 2012). Hence, replication is more competent with interfering satBaMV than BaMV and non-interfering satBaMV.

Both BaMV and satBaMV depend on BaMV RdRp for replication, so whether BaMV contains a similar AHSL structure in the 5′-UTR is of interest. The 5′-UTR RNA secondary structures of all natural BaMV isolates were analyzed by MFOLD but showed no secondary structure because of a highly repetitive sequence. The conserved AHSL in LSL was found only when the sequence extended to the ORF1 region (1-173 nt) (**Figure 1E**; Chen et al., 2012). This secondary structure of BaMV-S was confirmed by enzyme probing (Chen et al., 2010). As predicted, all analyzed BaMV isolates showed an identical AHSL structure with C^86^ in IL-1 and C^64^ in IL-2 regardless of whether satBaMV was associated with their replication or whether the associated satBaMV was interfering or non-interfering (Chen et al., 2012). The C^60^ in IL-2 and C^83^ in IL-1 of BSL6 (corresponding nucleotide C^86^ in IL-1 and C^64^ in IL-2 of BaMV) are essential for satBaMV-mediated BaMV interference (**Figure 1D**; Chen et al., 2007) and also important for BaMV replication (Chen et al., 2012). The BaMV-C^86^U mutant lost replication ability in *N. benthamiana* protoplasts and *C. quinoa*. The replication efficiency was reduced with BaMV-C^64^U–mutant infection as compared with BaMV infection alone. Thus, BaMV C^86^ is essential and C^64^ is important for BaMV replication. Furthermore, non-interfering satBaMV BSF4 could reduce the number of local lesions and BaMV-C^64^U level on co-infection in *C. quinoa*, so BSF4 may be more competent than BaMV-C^64^U for replication. In addition, increased level of BaMV was associated with reduced BSL6 level in the mixed inoculum (Chen et al., 2012). These results demonstrate that satBaMVs interfere with BaMV replication in a dose-dependent manner via replication complex competition.

## Trilateral Interaction Among BaMV, SatBaMV and Host Plants: Possible Involvement of RNA Silencing in SatBaMV-Mediated BaMV Interference

Interfering satRNAs attenuating HV-caused symptoms and reducing the HV level are complex interactions between the host plant, HV and satRNAs. However, the model of competition for replication complexes explains the interaction between only the HV and satRNAs. No other mechanisms were proposed and proven until large studies of RNA silencing and the generation of a large amount of next-generation sequence data from virus-infected samples. These “big” data reveal the trilateral interactions of host, HV and satRNAs. For example, small RNAs (sRNAs) of satRNAs (sat-sRNAs) can target HV and induce silencing of HV for CMV (Zhu et al., 2011); the satCMV of SD-CMV can reduce level of RNA-4A, which encodes the viral suppressor of RNA silencing 2b (VSR2b) protein, thereby diminishing the viral counter-defense strength by host immunity (Hou et al., 2011). In addition, Y satRNAs (Y-sat) of CMV can interfere in the function of VSRs by saturating the sRNA binding capacity of VSR (Shen et al., 2015). All this evidence shows that satRNAs take advantage of the host defense system and RNA silencing to interfere in HV replication.

RNA silencing is the major antiviral defense mechanism operating in a sequence-specific manner in plants (Ding, 2010). In general, double-stranded RNA formed during virus replication or the highly structured viral RNA can trigger RNA silencing by recognizing and dicing into 20- to 24-nt viral sRNAs (vsRNAs) by RNase III-like proteins, Dicer-like (DCLs) (Blevins et al., 2006). These vsRNAs are then recruited by ARGONAUTE proteins (AGOs) (Mallory and Vaucheret, 2010) and target the viral RNA or host genes with a complementary sequence. The viral RNAs or target genes would be cleaved and silenced by vsRNAs via RNA degradation. However, viruses also evolve to have the counter-defense mechanism by encoding a VSR. VSRs suppress RNA silencing by four major mechanisms. The most straight-forward and common way is by binding sRNAs. Second, they prevent the recognition and dicing of viral RNA by inhibiting DCLs. Third, they prevent the assembly of the RNA-induced silencing complex by targeting its components, such as AGOs. Finally, they inhibit the amplification of antiviral signals by interacting with RdRp or its interacting complexes (Burgyan and Havelda, 2011).

Satellite RNAs are both inducers and targets of RNA silencing. Highly structured satRNAs or satRNA-replication intermediate double-stranded RNAs induce RNA silencing and produce sat-sRNAs (Du et al., 2007; Lin et al., 2010). These sat-sRNAs can direct RNA cleavage of host genes (Shimura et al., 2011; Smith et al., 2011) or the HV genome (Zhu et al., 2011) and cause DNA methylation of host genes (Wang et al., 2001). However, unlike the HV, no satRNA encoded proteins were reported as VSRs. How interfering satBaMV manipulates the host RNA silencing immune system to reduce HV replication remains largely unknown, although strategies mediated by different satCMVs have been reported (Moriones et al., 1992; Hou et al., 2011; Shen et al., 2015). From small-RNA sequencing data, BaMV-derived sRNA (BaMV-sRNA) levels were not increased in BaMV and BSL6 co-infected samples, and no specific satBaMV-sRNAs of BSL6 could target BaMV genome (Lin et al., 2010). Although the 5′-UTR of BaMV contains a stretch of homologous sequence from nucleotides 1 to 30 (**Figure 1B**), BaMV-sRNAs and satBaMV-sRNAs of BSF4 and BSL6 generated from this region are extremely low in number (Lin et al., 2010). The sRNA hotspots within the 5′-UTR of BaMV and BSF4 located in the region from nucleotides 80 to 120 formed SLB and SLC and one strand of the stem region of SLC (Lin et al., 2010). Hence, RNA silencing may not be directly involved in satBaMV-mediated reduction in BaMV infection.

## Application of Interfering SatBaMV in BaMV Resistance

*Bamboo mosaic virus* infects more than 90% of bamboo plants with pachymorph rhizomes in Taiwan, which results in great economic loss (Lin and Chen, 1991; Lin et al., 1993). Because bamboo is usually vegetatively propagated, the use of indexed, non-infected bamboo generated from meristem tip culture as propagation materials would greatly improve BaMV disease control (Hsu et al., 2000). However, BaMV spread may be through unknown vectors, mechanical injury or contaminated tools used for propagation or harvesting. How to eliminate BaMV infection in healthy plants in the field is critical. One of the promising strategies is the use of virus-resistant cultivars.

Because satRNAs can attenuate HV-induced symptoms and/or reduce HV replication, they are good candidates as biological-control agents. In the late 1980s, satCMV transgenic plants showing CMV resistance were established despite the underlying mechanism remaining unknown (Harrison et al., 1987). Interfering satBaMV could attenuate symptoms and reduce the BaMV level in co-infected plants. Thus, transgenic plants expressing interfering satBaMV would be a feasible approach to alleviate infection with BaMV. In transgenic *N. benthamiana* expressing BSL6 satBaMV, two phenotypes were observed after BaMV infection: one group showed mild BaMV symptoms, and another group was symptomless (Lin et al., 2013). Moreover, BSL6 transgenic plants were resistant to both BaMV viral-RNA and virion infection and with better resistance to BaMV viral RNA than virion. The transgene, BSL6 replicon, was expressed at a relatively low level in transgenic lines but was highly induced after BaMV infection. Thus, highly inducing the transgene only after BaMV infection could avoid the highly expressed transgene-induced silencing in plant growth and development. Moreover, BSL6-transgenic plants are highly resistant whether under attack by BaMV viral RNA or virions. With all these features, interfering satBaMV-transgenic plants may be a good option for BaMV disease control.

RNA silencing may not be involved in the mechanism of satBaMV-mediated BaMV resistance in transgenic plants. Moreover, the plant innate immune system involving salicylic acid and jasmonic acid pathways was also not enhanced in satBaMV-transgenic plants. However, the resistance of satBaMV transgenic plants to BaMV was associated with the transgene expression level in transgenic lines under the mock condition. Non-replication satBaMV transgenic plants could not reduce BaMV replication (Lin et al., 2013). Thus, competition for replication complexes with BaMV is the possible mechanism in BaMV-resistant transgenic plants expressing interfering satBaMV.

## Perspectives

The AHSL secondary structure and two unique nucleotides (C^60^ and C^83^) of satBaMV 5′-UTR are critical for the interfering satBaMV reducing BaMV level and infection in plants. This AHSL structure and the critical nucleotide C in IL-1 is conserved in the BaMV 5′-UTR and also important for replication. Moreover, interfering satBaMV dose-dependently reduces BaMV level. Thus, interfering satBaMV-reduced BaMV level competes for the replication complex.

How a single nucleotide determines the interference ability of satBaMV deserves further investigation. Here we propose the possible underlying mechanism.

### Long-Distance RNA–RNA interaction

Viral RNAs are four-dimensional because of the complex tertiary interactions with the host and viral factors in specific viral infection stages. These long-distance RNA–RNA interactions control virus replication, translation and sub-genomic RNA transcription (Miller and White, 2006). Whether C^60^ in IL-2 and C^83^ in IL-1 interact with a terminal or internal element of satBaMV or BaMV critical for BaMV interference remains unknown. However, a BaMV chimeric mutant expressing the BSL6 5′-UTR driven by a sub-genomic promoter is sufficient to reduce both (+) genomic and sub-genomic RNA level without affecting (-) sub-genomic RNA level. As well, the reduced (+) genomic RNA level is greater than the (+) sub-genomic RNA level (Hsu et al., 2006). This result may imply that possible long-distance RNA–RNA interaction of satRNAs and BaMV affects only activation or assembly of an RdRp complex competent for (+)- but not (-)-strand synthesis.

### RNA methylation

Another hypothesis for a single nucleotide of satBaMV causing a great change in interference in BaMV-induced symptoms and BaMV replication is methylation of this specific nucleotide. Ribonucleotides are ubiquitously methylated in life at nitrogen, the oxygen of the 2′OH moiety at fifth-position carbon atoms in pyrimidine, and second- and eighth-position carbon atoms in adenosines (Motorin and Helm, 2011). Methylated cytosine (m^5^C) is the most privileged. Cytosine can be easily transformed into uracil via deamination. However, m^5^C cannot be converted to uracil. Cellular RNAs containing m^5^C include transfer RNA (tRNA), ribosomal RNA, mRNA and non-coding RNA in both eukaryotes and prokaryotes (Squires et al., 2012; Edelheit et al., 2013; Hussain et al., 2013; Burgess et al., 2015; Delatte et al., 2016). Also, m^5^C was found in some animal viruses (Dubin and Stollar, 1975; Sommer et al., 1976). M^5^C is important for stabilization and Mg^2+^ binding of tRNA (Basti et al., 1996; Stuart et al., 2003; Helm, 2006), translation of mRNA (Strobel and Abelson, 1986) and weakening stimuli to the human innate immune system (Kariko et al., 2005). In adenovirus-infected HeLa cells, m^5^C was found only in adenovirus RNA (Sommer et al., 1976) but not mRNA (Furuichi et al., 1975; Salditt-Georgieff et al., 1976). As well, the tRNA-like structure of an isolate of *Turnip yellow mosaic virus* injected into *Xenopus* oocytes could be methylated at cytosine (Brule et al., 1998).

How viral RNAs are specifically methylated and the biological function of m^5^C in viral RNA needs further study. Here, we propose two hypotheses. One is that m^5^C^60^ and m^5^C^83^ may appropriately and efficiently dock into the active site of key factors of replication complexes. Alternatively, the methylation of cytosine in the tRNA-like structure of BaMV 3′-UTR may be critical for the interaction between replication complexes, BaMV 5′-UTR and 3′-UTR, and this interaction may be affected by the interfering satBaMV 5′-UTR during replication, thus reducing BaMV replication at both the (+)- and (-)-strand level. Bisulfite sequencing (Schaefer et al., 2009) could be used to elucidate whether C^60^ and C^83^ of satBaMV and C of BaMV 3′-UTR are methylated or not. However, the biological function of these methylated satBaMVs on BaMV replication is difficult to prove. A putative methyltransferase was found to interact with BaMV RdRp and suppress BaMV replication (Cheng et al., 2009). The involvement of RNA m^5^C methyltransferases in satBaMV-mediated BaMV interference is worthy of further investigation.

### Host factors or miRNAs involved

Whether specific host factors are recruited by interfering satBaMV for interference remains unknown but could be tested by comparing the protein profiles bound to the 5′-UTR of BSF4 and BSL6. The specific AHSL-interacting proteins can be detected by using the 5′-UTR of BSF4 and BSL6 as probes, followed by mass spectrometry identification.

Moreover, sRNA sequencing and array analysis revealed that the plant innate immune system is not involved and RNA silencing may not be directly involved in the mechanism of satBaMV-mediated BaMV interference. However, interfering satBaMV-induced specific microRNAs (miRNAs) or specific satBaMV-sRNAs may likely target the host gene, which is important for BaMV replication or essential effectors of the host innate immune system other than RNA silencing. Thus, the involvement of RNA silencing in BSL6-mediated interference remains an open question. It could be evaluated by using plant mutants defective in key components of RNA silencing or plants overexpressing VSRs and further analyzing satBaMV-induced specific satBaMV-sRNAs, miRNAs and other types of host endogenous small RNAs. The mechanism underlying interfering satBaMV reducing BaMV level and host symptom development remains a fascinating question requiring long-term study.

## Author Contributions

Drafting the article: K-YL, Critical revision of the article: N-SL and final approval of the version to be published: K-YL and N-SL.

## Conflict of Interest Statement

The authors declare that the research was conducted in the absence of any commercial or financial relationships that could be construed as a potential conflict of interest.
